# Midwifery tutors' capacity and willingness to teach contraception, post-abortion care, and legal pregnancy termination in Ghana

**DOI:** 10.1186/1478-4491-8-2

**Published:** 2010-02-23

**Authors:** Gertrude Voetagbe, Nathaniel Yellu, Joseph Mills, Ellen Mitchell, Amanda Adu-Amankwah, Koma Jehu-Appiah, Felix Nyante

**Affiliations:** 1 Ipas Ghana, PMB CT 193 Cantonments, Accra, Ghana; 2 Research and Development Division, GHS, PO Box MB190, Accra, Ghana; 3 Ipas, Chapel Hill, North Carolina, USA; 4 Nursing and Midwifery Council, Accra, Ghana

## Abstract

**Background:**

Ghana has a high maternal mortality rate of 540 per 100 000. Although abortion complications usually are treatable, the risks of morbidity and death increase when treatment is delayed. Delay in care may occur when women have difficulty accessing treatment because health care providers are not trained, equipped, or willing to treat the complications of abortion. Gaps in the midwifery tutors' knowledge on comprehensive abortion care (CAC) have resulted in most midwives in Ghana not knowing the legal indications under which safe abortion care can be provided, and lacking the skills and competencies for CAC services. The aim of this study is to assess the capacity and willingness of midwifery tutors to teach contraception, post abortion care and legal termination in Ghana.

**Methods:**

This study focused on all 14 midwifery schools in the country. A total of 74 midwifery tutors were interviewed for this study. Structured self-administered questionnaires were used for data collection. The data were entered and checked for consistencies using Epiinfo 6.04 and analyzed using Stata 8. Descriptive analysis was used and frequencies reported with percentages.

**Results:**

In total, 74 midwifery tutors were interviewed. Of these, 66 (89.2%) were females. The tutors had mainly been trained as midwives (51.4%) and graduate nurses (33.8%). Respondents were predominantly Christians (97.3%).

The study discovered that only 18.9% of the tutors knew all the legal indications under which safe abortion care could be provided. The content of pre-service training of tutors did not include uterine evacuation with manual vacuum aspirator (MVA).

The study also highlighted some factors that influence midwifery tutors' willingness to teach comprehensive abortion care. It was also revealed that personal and religious beliefs greatly influence teaching of Comprehensive Abortion Care.

**Conclusion:**

The findings of this survey suggest that the majority of tutors did not know the abortion law in Ghana as well as the Ghana Health Service Reproductive Health Standards and Protocol. Thus, there is a need to enhance their capacities to teach the present pre-service students the necessary skills to offer CAC after school and to understand related issues such as related legal matters.

## Background

According to the Ghana Medical Association, unsafe abortion is the second highest contributor to the country's maternal mortality ratio of 540 deaths per 100 000 live births [1]. A case review of hospital admissions during the calendar year 2000 at the Korle-Bu Teaching Hospital (Ghana's largest teaching hospital) found that 41% of admissions were due to complications related to abortion [2]. Although most abortion complications are treatable, the risk of morbidity and mortality increases when treatment is delayed [3]. Delay in care may occur when women have difficulty accessing treatment because health care providers are not trained, equipped, or willing to treat the complications of abortion.

Most skilled health professionals capable of managing abortion complications remain in urban areas. In the rural areas, midwives are the main service providers, therefore preparing them to provide comprehensive abortion care is critical [3-5]. Studies have shown that with adequate training and clinical hands-on practice, midlevel providers can offer uterine evacuation [6].

Gaps in the midwifery tutors knowledge on Comprehensive Abortion Care (CAC) have resulted in most midwives in Ghana not knowing the legal indications under which safe abortion care can be provided as well as lacking the skills and competencies for CAC services.

In Ghana, strategies to address this gap have been largely limited to in-service approaches [4,7,8]. Pre-service training has been more limited. Overall, about 500-600 midwives graduate from the various types of midwifery programs each year. Despite the number of midwives who pass out of the schools, few midwives are available to provide reproductive health needs in the public sector due to the brain drain. Prior to this study, the standard midwifery curriculum that was used to train the tutors included post abortion care (PAC) and contraception, but it excluded other components of comprehensive abortion care such as options counseling and legal termination.

Social, religious and cultural beliefs of midwifery tutors may influence their attitudes towards teaching comprehensive abortion care [7,9]. International support for increasing midlevel providers' role in abortion care is evident in statements and guidance from influential organizations, including medical associations and coalitions. In 1990, a statement jointly endorsed by the International Confederation of Midwives (ICM), the World Health Organization (WHO) and United Nations Children's Fund (UNICEF) called for countries to incorporate training in emergency uterine evacuation into midwifery education, in the context of their effort to promote safe motherhood [10].

At the time of this study, the authors were unaware of any study conducted to comprehensively explore midwifery tutors' knowledge concerning CAC, nor their ability and willingness to teach topics concerning abortion.

To examine the feasibility of expanding midwifery schools' curricula, the Ghana Ministry of Health, the Ghana Registered Midwives Association, Population Council, JHPIEGO and Ipas implemented a comprehensive operations research project to identify the ability and knowledge gaps with respect to comprehensive abortion care services and also to explore the willingness of midwifery tutors to offer clinical training in contraception, post abortion care and legal pregnancy termination.

Specifically, this study focuses on the capacity and willingness of midwifery tutors to teach contraception, post abortion care and legal termination.

## Methods

This exploratory study focused on all 14 midwifery schools in Ghana. Table 1 shows the list of midwifery schools in Ghana. Data were collected in February 2007 using a structured self-administered questionnaire which included questions on the following topics:

• Knowledge of law on abortion in Ghana

• Educational preparation/content of pre-service training

• Personal beliefs versus professional responsibility

• Motivational factors for teaching comprehensive abortion care

• Inhibiting factors to teaching comprehensive abortion care

Knowledge of the law on abortion in Ghana and educational preparation in terms of the content of the tutors' pre-service training were used as indicators to assess the midwives' capacity to teach CAC. In exploring their willingness, questions were asked on motivational and inhibiting factors to teach CAC as well as their personal beliefs versus professional responsibility.

**Table 1:** List of midwifery training schools in Ghana, 2007

SCHOOL	LOCATION
Korle-Bu Public Health Nurses Training	Accra

Korle-Bu Midwifery Training	Accra

37 Midwifery Training	Accra

Hohoe Midwifery Training	Volta Region

Koforidua Midwifery Training	Eastern Region

Atibie Midwifery Training	Eastern Region

Kumasi Midwifery Training	Ashanti Region

Mampong Midwifery Training	Ashanti Region

Offinso Midwifery Training	Ashanti Region

Berekum Midwifery Training	Brong Ahafo Region

Sekondi Midwifery Training	West Region

Cape Coast Midwifery Training	Central Region

Jirapa Midwifery Training	Upper West

Bolgatanga Midwifery Training	Upper East

A total of 123 tutors listed from all the midwifery schools were selected for the interview. Tutors at the midwifery schools are either classified as part-time or full-time. However, for the purpose of this study no distinction was made between the two categories of tutors, since they teach the same curriculum. For those who were either not readily available or present during the period of data collection, questionnaires were left at the schools to be given to them. As is the case with self administered questionnaires, some tutors did not return the questionnaires. In all, 74 out of the 123 selected tutors completed the questionnaires, yielding a response rate of 60.2%. The non respondents were mainly the part-time tutors. It is important to state that responses were obtained from tutors from all the midwifery schools in Ghana.

The data were entered and checked for consistencies using Epiinfo 6.04 and analyzed using Stata 8. Descriptive analysis was used and frequencies reported with percentages. Scores were matched in order to ascertain the midwives' knowledge of the legal indications under which CAC is permissible in Ghana.

## Results

### Socio-demographic characteristics of midwifery tutors

A total of 74 midwifery tutors were interviewed. Out of these, 66 (89.2%) were females and 8 (10.8%) were males. Most tutors were within the age group of 50-59 years (33.8%). Respondents were predominantly Christian (97.3%). The tutors had mainly been trained as midwives (51.4%) and graduate nurses (33.8%).

### Knowledge of the law on abortion in Ghana

In Ghana, safe abortion is permitted by law under certain conditions. These are:

i. It must be performed by trained, qualified medical practitioner;

ii. It must be in a registered health facility; and

iii. It must be in accordance with one at least one of the legal conditions under which abortion is permissible. These are:

• Pregnancy as a result of rape or defilement;

• Pregnancy as a result of incest;

• Continuance of pregnancy will involve risk to life of the pregnant woman;

• Continuance of pregnancy will involve risk or injury to her physical health;

• Continuance of pregnancy will involve risk or injury to her mental health;

• Where there is substantial risk that the child, if born, may suffer from or later develop a serious physical abnormality or disease; and

• Where the woman is mentally subnormal or mentally challenged.

**Figure 1:**
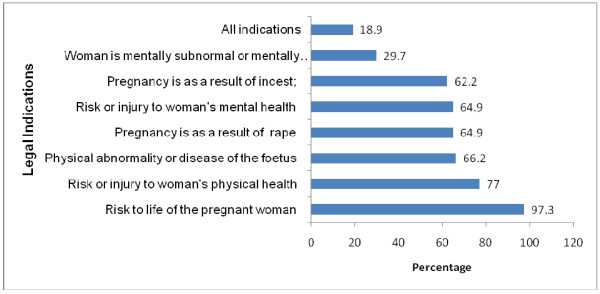
**Midwifery tutors' knowledge of the legal indications under which abortion is permissible in Ghana, 2007**.

The provision of safe abortion care must be in conformity with the Ghana Health Service Standards and Protocols. Figure 1 represents midwifery tutors' knowledge of the legal indications under which CAC is permitted.

Overall, the knowledge of the circumstances under which safe abortion care can be provided was low among tutors (18.9%). However, 97.3% correctly identified that if the pregnancy involves risk to the life of the woman, safe abortion is permitted. Only 29.7% of the tutors were aware that abortion is legal when the pregnant woman is mentally subnormal or mentally challenged.

### Educational preparation and content of pre-service training

Information about the tutors' educational background and the number of years they had been teaching was ascertained. The number of years of teaching ranged from less than 1 year to 37 years, with a median of 12 years.

Pre-service training of tutors of midwifery schools did not include all the methods of abortion. As shown in Table 2, the pre-service training of 77% of tutors did not include uterine evacuation with Manual Vacuum Aspiration (MVA), 73% indicated that their training did not include information about MVA instruments. Seventy-three percent said their training did not include medication abortion. The tutors indicated that pain management for uterine evacuation (51.4%), abortion counselling (47.3%) and confirming completeness of abortion (51.4%) were not covered in their training.

**Table 2:** Elements of tutors' pre-service training reported by 74 midwifery tutors in Ghana, 2007

Categories	%
**Gestational dating**	
Last menstrual period	90.5
Bimanual exam	52.7
Ultrasound	18.9
**Contraception**	
Short-term methods	91.9
Intrauterine device insertion (IUCD)	77.0
Tubal ligation	48.6
**Counselling**	
Abortion counselling	52.7
Post abortion counselling	59.5
**Uterine evacuation**	
D&C	36.5
MVA	23.0
Medication abortion	27.0
**Others**	
Infection prevention	91.9
Management of incomplete abortion	77.0
Referral of abortion complications	68.9
Community to prevent unsafe abortion	51.4
Pain management for uterine evacuation	48.6
Confirming completeness of an abortion	48.6
MVA instrument facts and features	27.0
Monitoring quality of abortion services	17.6
Ghanaian Abortion laws & GHS policies	25.7

However, the respondents had training in management of incomplete abortion (77.0%), referrals of abortion complications (68.9%), short-term contraceptive methods (91.9%) and gestational dating via bimanual (52.7%).

Although the midwifery tutors interviewed had some training in CAC, it was revealed that none of them had clinical skills training.

### Personal beliefs versus professional responsibility

Health care providers bring personal views and values in discharging their professional duties. The study revealed that 18.9% of the tutors found the issue of abortion as permitted by law personally objectionable. More than a third (37.8%) mentioned abortion to be contrary to their religious beliefs. More than a tenth (12.2%) indicated that they were not likely to teach it because their friends were opposed to the provision of abortion care. Only 9.5% of the tutors were worried about their reputation in society. Seventy percent of the tutors interviewed stated that when abortion services are made readily available for pregnant women and girls who were raped, many will claim to be raped even when it is not the case.

### Motivational factors for teaching Comprehensive Abortion Care

Several factors influence midwifery tutors' willingness to teach Comprehensive Abortion Care. It was clear from the survey that most tutors teach abortion under management of PAC and infection prevention rather than provision of CAC as permitted by law. Three common motivational factors cited by midwifery tutors as influencing their willingness to teach CAC were: the desire to teach their students to be able manage injuries that result from self induced abortion (82.4%), the desire to help students, after graduation, to be able to reduce maternal death and disabilities in Ghana by providing quality CAC services (79.7%) and the desire to provide comprehensive training for students (75.7%).

### Inhibiting factors to teaching Comprehensive Abortion Care

Midwifery tutors surveyed expressed some concerns for teaching CAC. The most frequent factor mentioned for hesitation was uncertainties about circumstances under which the law permits abortion (60.8%). This was followed by legal problems (39.2%) and religious conflicts (37.8%). Uncertainties about the policies and procedures for teaching safe abortion as well as clinical competencies were mentioned (36.5%). Less than one-fifth of the tutors were concerned about lack of support from school administration.

## Discussion

The provision of safe abortion care by trained health professionals is governed by policies and protocols of the Ghana Health Service (GHS) which has the mandate of overseeing all public health issues. In 2003, the Ministry of Health and GHS revised the National Reproductive Health Policy to include PAC and the provision of CAC as permitted by law [11]. The 2006 GHS Reproductive Health Standards and Protocol provide guidelines for interpreting the law and these are consistent with the World Health Organization's guidelines and Standards of Best Practice [12]. Of all the 74 midwifery tutors who were surveyed, only 18.9% were aware of all the legal indications under which safe abortion is permitted. About three quarters of the tutors (74.3%) mentioned that their pre-service training did not include Ghanaian abortion Law and GHS policies. This technically limits access to abortion care since providers who do not understand the abortion law in the country may refuse to provide legal abortion services as well as teach it. It is useful to know however that most tutors learn of the circumstances under which legal abortion care can be provided after their training. It is therefore important that midwifery tutors are knowledgeable in the policies and law, given that midwives are the main service providers in rural communities, which constitute over 60% of Ghana's population, and may experience limited opportunities for continuous education [4].

The components of Comprehensive Abortion Care (CAC) are options counselling, induced abortion, post abortion care and post abortion contraception. These are considered as advance skills which are acquired through in-service trainings. This means that newly trained midwives will not be able to effectively provide quality abortion services and provide the necessary care or refer to a higher level facility if necessary. Studies have shown that with adequate training and clinical hands-on practice, midlevel providers can offer uterine evacuation with MVA [6].

The lack of training in uterine evacuation means that midwives are only limited to certain specific skills such as delivery, though it is important that they are trained in the use of appropriate methods for the management of abortion care. Midwives receiving the pre-service training had a higher knowledge of family planning methods and were more likely to provide information on method specific side effects during counselling [13].

The study highlighted some reasons why midwifery tutors are hesitant to teach CAC. These included:

• uncertainties about circumstances under which the law permits abortion;

• legal problems;

• religious biases;

• uncertainties about the policies and procedures for teaching safe abortion; and

• uncertainties about their clinical competencies.

Social and religious beliefs of health professionals play an important role in the provision of health care service delivery. Ideally, personal beliefs should not influence the care a client seeking abortion receives. However, ethical, religious and cultural values influence the teaching and provision of abortion services as granted under the Ghanaian law.

## Conclusion

The findings of this study show that the majority of the tutors do not completely know all the circumstances under which safe abortion care can be provided. Given that the provision of abortion care is governed by PNDC Law 102 [14], it is important that abortion care is included in the curriculum of midwifery tutors' training. This will equip midwifery tutors with the necessary knowledge to teach the student midwives to be able to provide safe abortion services. This is crucial as over 60% of the country's population live in rural areas where midwives are the main service providers.

The curriculum of midwifery tutors' training should be expanded to include the various methods of providing safe abortion care. Presently, it is clear that the curriculum of midwifery tutors does not include certain aspects and methods of abortion care. If the curriculum is expanded it will enable tutors to teach midwifery students all the methods of abortion care in their appropriate context. It will also let them ensure that their students understand the laws and policies governing abortion care in Ghana.

The findings clearly show that personal beliefs greatly influence the teaching of abortion care in midwifery training schools. Efforts should therefore be made to educate tutors on the teaching of abortion care as provided by law. Distinction should be made between professional responsibilities and personal beliefs in making a decision to teach or not to teach abortion care.

Regular training sessions should be held for tutors of midwifery schools on the various methods of providing comprehensive abortion care to update them and build their capacity to teach topics on abortion and related issues. Training guidelines should be prepared and disseminated to tutors of midwifery training schools for effective teaching and learning. Opportunities should be created for tutors to gain additional training on a regular basis. This will motivate them to teach and provide Comprehensive Abortion Care.

The findings of this survey suggest that the majority of tutors did not know about the abortion law in Ghana as well as the GHS Reproductive Health Standards and Protocol. Therefore, there is the need to enhance their capacities to teach the present pre-service students the necessary skills to offer CAC and understand the relevant laws and other related issues.

## Abbreviations

CAC: Comprehensive Abortion Care; D&C: Dilatation And Curretage; GHS: Ghana Health Service; ICM: International Confederation Of Midwives; MVA: Manual Vacuum Aspiration; NMC: Nurses And Midwives Council; PAC: Post Abortion Care; SAC: Safe Abortion Care; UNICEF: United Nations Children's Fund; WHO: World Health Organization.

## Competing interests

The authors declare that they have no competing interests.

## Authors' contributions

GV was the lead contributor of this manuscript. NY participated in analysing the data and assisted to draft as well as reviewing the manuscript. EM conceived the study, and helped to draft manuscript. FN, and AA were involved in the data collection and drafting as well as reviewing the manuscript. JM and KJ have been involved in the drafting and critical revision of the manuscript. All authors read and approved the final version of the manuscript.
